# Acknowledgment to Reviewers of *Entropy* in 2020

**DOI:** 10.3390/e23020144

**Published:** 2021-01-25

**Authors:** Entropy Editorial Office

**Affiliations:** MDPI AG, St. Alban-Anlage 66, 4052 Basel, Switzerland

Peer review is the driving force of journal development, and reviewers are gatekeepers who ensure that *Entropy* maintains its standards for the high quality of its published papers. Thanks to the cooperation of our reviewers, in 2020, the median time to first decision was 17 days and the median time to publication was 38 days. Among the reviewers who reviewed for *Entropy* in 2020, the following five have been selected to receive the “*Entropy* 2020 Outstanding Reviewer Award” for the timeliness and quality of their review reports in 2020:

Ciuonzo, Domenico from University of Naples Federico II, Italy
Keshmiri, Soheil from Advanced Telecommunications Research Institute International, Japan
Tseng, Chih-yuan from University of Alberta, Canada
Bajic, Dragana from University of Novi Sad, Serbia
Rangel-Hernandez, Victor Hugo from University of Guanajuato, Mexico

The editors would like to express their sincere gratitude to all of the following reviewers for their precious time and dedication, regardless of whether the papers were finally published:
Aavani, AmirLim, HyunkiAbah, ObinnaLima, Leonardo Dos SantosAbbas-Aghababazadeh, FarnooshLin, AijingAbbruzzo, AntoninoLin, HaijinAbdel-Aty, MahmoudLin, Han-YuAbdel-Latif, AhmedLin, Hsuan-YinAbdellatif, MohamedLin, Hsueh-chunAbdelmaguid, TamerLin, JunLinAbdelsalam, Sara I.Lind, Pedro G.Abdo, HanyLing, BoAbebe, AmareLinial, MichalAbegão, LuisLinninger, Andreas A.Abellán, JoaquínLio, YuhlongAboutorab, NedaLipic, TomislavAbreu, Everton M.C.Lipowski, AdamAbyzov, Alexander S.Little, Daniel R.Adamson, MatthewLittle, Douglas J.Adrangi, BahramLittle, Roderick J.A.Afify, AhmedLiu, AnqiAgarwal, ChiragLiu, Chia-ChiAgarwal, PraveenLiu, Chia-HuiAgarwal, RaviLiu, GuilongAghajani, HalehLiu, HanliAgnesi, CostantinoLiu, Hsiao-ChuanAguerri, Inaki EstellaLiu, Jinn-LiangAguilar-González, AbielLiu, Kang K.L.Aguilella-Arzo, MarcelLiu, ShuaiqiAhmad, JawadLiu, XinfengAhmad, MusheerLiu, ZhengjunAhmad, RaufLivieris, Ioannis E.Ahmadi, MohammadLizcano Casas, DavidAhmadian, AliLizier, JosephAhmed, S. EjazLlenos, AndreaAhmed, UmairLo Franco, RosarioAidoo, AnthonyLobachev, OlegAiordachioaie, DorelLobo, Iarley PereiraAkilan, ThangarajahLocatelli, EmanueleAkiyama, YoshitsuguLoeffler, TroyAkkermans, RinieLogan, Robert K.Akram, UmairLok Woo, WaiAlabrudziński, SławomirLollai, Stefano A.Alaee-Kerahroodi, MohammadLong, G.L.Alamaniotis, MiltiadisLong, Gui-LuAlanis, Alma Y.Longtin, AndréAlarcon-Aquino, VicenteLoperfido, NicolaAlava, MikkoLopes, António M.Albantakis, LarissaLopes, WaslonAlberti, TommasoLópez Ramos, Juan AntonioAlbertini, Bruno De CarvalhoLópez, CristóbalAlbonico, AliceLópez, RobertoAlbrecher, HansjoergLópez-Estrada, Francisco RonayAlbu, FelixLópez-Hernández, Fernando A.Alejo Eleuterio, RobertoLópez-Mancilla, DidierAlemneh, Daniel GelawLópez-Martínez, Jesús AlbertoAleroev, TemirkhanLopez-Monsalvo, Cesar S.Alessandri, AngeloLópez-Pascual, JoaquínAlessandro, TrevesLorenz, RobinAlexan, WassimLoskot, PavelAlexandru, CătălinLostado, RubénAlexey, KanelLotric, UrosAlfarano, SimoneLou, Der-ChyuanAlfaro-Ayala, Jorge ArturoLoubenets, Elena R.Alfian, GanjarLourenço Nunes, Paulo JorgeAli, FarmanLouzada, FranciscoAli, MusrratLow, RandAlicea, BradlyLozi, RenéAllani, SabriLu, BenzhuoAllen, Gayle DonaldLu, Chi-JieAllen, Jeffrey S.Lu, Ching-TaAllili, Mohand SaidLu, YingdongAl-Mdallal, Qasem M.Lubý, ŠtefanAlmeida, CaioLuca, AdrianAlmeida, ThiagoLucchini, MaristellaAl-Naji, AliLucia, UmbertoAlodat, TareqLučić, BonoAlonso-Conde, Ana BelénLudovico, Maria FlorenciaAl-Rifaie, Mohammad MajidLuin, StefanoAl-Shargie, FaresLundow, Per HåkanAlsharif, Mohammed H.Luo, AlbertAltaisky, MikhailLuo, Win-jetAlvaredo, PaulaLuque, BartolomeAlvarez, EstrellaLuque, JoaquínAly Abdallah, Abdelraheem MahmoudLushington, GerryAly, KarimLuukka, PasiAlzaatreh, AymanLuzzi, LauraAlzabut, JehadLynn, Christopher W.Amancio, DiegoLynn, TheoAmero, CarlosMa, XiongfengAmico, EnricoMaarten, GrachtenAmigó, José MaríaMachado, PedroAmosov, GrigoriMachta, BenjaminAmpeliotis, DimitrisMachta, JonAnand, PrashantMachuca, Juan L. MataAnastassopoulos, VassilisMachura, LukaszAndai, AttilaMacias-Diaz, Jorge E.Andres, EmmanuelMaciel, CarlosAndriyanova, IrynaMaciel, LeandroAndrzejczyk, RafałMaddamsetti, RohanAng, Chee WeiMady, Carlos Eduardo KeutenedjianAngel, LuisMagafas, LykourgosAngelo, Renato M.Magazzu, LucaAnghel, Dragos-VictorMagee, ChrisAngiulli, GiovanniMagenes, GiovanniAngstmann, ChristopherMagg, SvenAngulo, José M.Maggi, MarioAngulo-Brown, FernandoMahian, OmidAnishchenko, LesyaMahmoud, G.M.Anitas, Eugen MirceaMahowald, KyleAnnen, JitkaMaietta, SaverioAntonio, IovanellaMainardi, FrancescoAntonio, Stella FabioMaistro, MariaAntonopoulos, AngelosMajee, ArghyaAnwar, Mehdi F.Majka, AndrzejAo, PingMakantasis, KonstantinosApfelbaum, Evgeny M.Makarov, Valeri A.Aprea, CiroMakieła, ZbigniewAquilanti, VincenzoMakowiec, DanutaAragoneses, AndrésMakowski, MarcinAramendi, ElizabeteMakris, ChristosArandjelovic, OgnjenMalan, KatherineAraya, GuillermoMalarz, KrzysztofArbel, JulyanMalecka, JoannaArbuzov, AndreiMalek-Madani, RezaArdenghi, Juan SebastiánMalgaretti, PaoloArgyrakis, PanosMalik, NishantArima, TakashiMallory, Stewart A.Arizmendi, MiguelMalmström, LarsArkhipov, Ievgen I.Malone, DavidArnous, Ahmed H.Maluf, José WadihArora, SiddharthMalyarenko, Anatoliy A.Aros, RodrigoManana-Rodriguez, JorgeArraut, IvanMancino, LucaArsac, Laurent M.Mandelli, StefanoArtur, DabrowskiMandrikova, OksanaArunachalam, ShivaramMangioni, GiuseppeAsghar Heidari, AliManis, GeorgeAshcroft, GrahamManneville, PaulAsif, RameezManss, ChristophAslam, MuhammadMantzaris, Alexander V.Aslam, SherazManzano, DanielAsllani, MalborManzo, MarioAssad, MamdouhMao, XiaoanAssad, SyedMaragakis, MichaelAssenza, GiovanniMaragkoudakis, ManolisAssis, Karcius Day RosárioMarateb, Hamid RezaAtanackovic, TeodorMarchetti, DedaloAtman, AllbensMarcon, EricAttux, RomisMarcos, José DanielAur, DorianMarcy, RichardAusloos, MarcelMarensi, ElenaÁvalos, Juan GerardoMarfatia, HardikAvendaño, Alejandro CardenasMarinazzo, DanieleAvila, MarcMarini, MartinoAyers, PaulMarkelov, Oleg A.Azam, Naveed AhmedMarkov, KonstantinAzevedo, AlcinoMarković, DimitrijeAziz, WajidMarkovič, ReneBabaie Mahani, AlirezaMarkus, FerencBabenko, MikhailMarkussen, BoBabii, AndriiMarle, Charles-MichelBacardit, JaumeMarolt, MatijaBaccala, Luiz AntonioMarović, IvanBacco, DavideMarquez, JaimeBaczynski, DariuszMarsh, StephenBadawi, AymanMarsiglietti, ArnaudBadcock, PaulMaršík, FrantišekBae, JoonwooMarsland III, RobertBae, YoungchulMartens, CraigBagnato, Vanderlei SalvadorMartin, LuciBai, BaomingMartin, Randi C.Bai, BofengMartinez, Alexandre SoutoBaig, ImranMartinez, AlfonsoBailey, JosephMartinez, Harkaitz EguiraunBaïri, AbderrahmaneMartínez-Hinarejos, Carlos-D.Bajic, DraganaMartínez-Ratón, YuriBajić, DragoljubMartínez-Val, J.M.Bakaev, MaximMartinez-Valdes, EduardoBakhtin, VictorMartino, AlessioBalasubramanian, KrishnanMartino, José Mario DeBamba, KazuharuMartino, LucaBanasiak, JacekMartinopoulos, GeorgiosBancal, Jean-DanielMartins, DianaBanerji, SugataMartins-Filho, CarlosBania, PiotrMartyushev, LeonidBanik, IndranilMaruschak, PavloBao, BochengMary, PhilippeBarbancho, AnaMarzen, SarahBarbaresco, FredericMaskeliunas, RytisBarbieri, NilsonMason, JayBarbon Junior, SylvioMassuça, Luís MiguelBarbosa, Anderson L.R.Mastrangeli, MassimoBarbu, Vlad StefanMastriani, MarioBardera, AntonMastroeni, LorettaBardi, UgoMastropietro, AlfonsoBarghathi, HatemMathwig, KlausBariviera, Aurelio FernándezMatouk, Ahmed EzzatBarletta, LucaMatsoukas, ThemisBarquero-Pérez, ÓscarMatsubara, HirokiBarra, FelipeMatsuda, IchiroBarranco-Jiménez, MarcoMatsumoto, RyutarohBarreto, Guilherme A.Matsumura, KentaBarriuso, Alberto L.Matsunaga, YasuhiroBarros, MárciaMatsushima, ToshiyasuBartels, DavidMatsuzaki, KentaroBartkowiak, TomaszMatthiesen, BhoBartlett, StuartMattingly, HenryBartnicki, JarosławMauleón, IgnacioBartok-Partay, LiviaMauri, RobertoBartolacci, MichaelMausbach, PeterBasaran, CemalMavrantzas, VlasisBasios, VasileiosMayer, ChristopherBassi, GermánMaynar, PabloBastarrachea-Magnani, Miguel AngelMazet, VincentBastieri, DenisMazur, StepanBasto, MárioMazurek, MiroslawBasu, AyanendranathMazurek, PrzemysławBasu, IshitaMazziotti, DavidBasu, PrabirMcCabe, BrendanBasu, SumantaMcCamley, John D.Batista Henriques, IzabelaMcCash, LuthaisBatiste, OriolMcGarr, Arthur F.Batsidis, ApostolosMcRoberts, NeilBauchy, MathieuMediano, Pedro A.M.Baudot, PierreMedina, Jose M.Bauer, RomanMeena, Nand KishorBaumann, Michael HeinrichMehmood, RashidBautu, ElenaMeichanetzidis, KonstantinosBaxter, GarethMeisen, TobiasBayarri, Miguel SoutoMejía, AndrésBayat, AbolfazlMejía, Carlos E.Be’ery, YairMejía-Salazar, Jorge RicardoBebiano, NatáliaMelbourne, JamesBecerra, ArturoMelkikh, Alexey V.Becerra, Miguel A.Melle-Franco, ManuelBechtel, WilliamMelnikov, Alexey A.Beckwith, AndrewMena, GonzaloBehe, MichaelMendes, JérômeBeims, Marcus W.Mendez, FranciscoBeklaryan, ArmenMendl, Christian B.Belaid, AbdelMendonça, José Ricardo G.Bełdowski, PiotrMenéndez, Héctor D.Belhaj, AdilMeo, MariannaBellini, AlbertoMercier, DavidBellomo, GuidoMesa, OscarBelman-Flores, Juan M.Mesjasz, CzesławBelonenko, MikhailMessina, AntoninoBelov, AlexanderMetzler, RalfBelyaev, EvgenyMiao, HongyuBen Jabeur, SamiMichaelian, KaroBen Sherwood, BenMichail, TsagrisBenato, AlbertoMichel, FlorentBendechache, MalikaMichelitsch, ThomasBenet, LuisMiedzińska, DanutaBenioff, PaulMieloszyk, MagdalenaBenish, William A.Migallón, HéctorBenítez Andrades, Jose AlbertoMihai, AndruscaBenka, PavelMiklavcic, StanBen-Naim, AriehMiksovsky, JiriBen-Yishai, AssafMikulka, JanBenyoucef, AbdelghaniMilani, AlfredoBerend, DanielMills, DeEttaBereyhi, AliMilosevic, MiloradBereziński, PrzemysławMimoso, José P.Berezowski, MarekMinussi, Carlos RobertoBerezvai, SzabolcsMiolane, LeoBerger, SebastianMiolane, NinaBerillis, PanagiotisMiquel, Paul-AntoineBeris, AntonyMiranda, Jose Garcia VivasBernaola-Galvan, Pedro A.Miranda, Maria D.Bernardes, Nadja KolbMircea, Sasu LucianBernardino, JorgeMiret-Artés, SalvadorBernaś, MarcinMirjalili, SeyedaliBernhauerová, VeronikaMirkes, Evgeny M.Bernroider, GustavMishra, AmritaBerry, TyrusMishura, YuliyaBerthoumieu, YannickMiskiewicz, JanuszBertin, EricMisra, AvijitBessa, Iury ValenteMistiaen, WilhelmBesteiro, Lucas VazquezMiszczak, Jaroslaw AdamBetti, SilvelloMitroi-Symeonidis, Flavia-CorinaBhattacharya, TanmoyMitrović Dankulov, MarijaBhuiyan, EhsanMlotkowski, WojciechBiagi, Pier FrancescoMöckl, LeonhardBiałek-Jaworska, AnnaModanese, GiovanniBianco, GiuseppeMohamed, SharmarkeBianco, ValentinoMohammad, Ali Javaheri JavidBiçer, YusufMohammadi, BabakBielecka, MarzenaMohan, Chilukuri K.Bielecki, AndrzejMohtashsami Borzadan, Gholam R.Bier, MartinMolares-Vila, AlbertoBigio, Irving J.Molina, VicenteBILAL, MuhammasMoll, Henri C.Billiard, SylvainMonaco, AlfonsoBinder, PhilippeMonica, CosteaBingert, SvenMontalvo, SotoBinois, MickaelMontani, FernandoBiondo, Alessio EmanueleMontecchi, LeonardoBisogno, MarcoMontemayor, CarlosBiswas, SoumyajyotiMontero, José-MaríaBizarro, João P.S.Monterroso-Checa, AntonioBlakely, Jonathan N.Montoya, Alejandro Raúl HernándezBlanc, WilfriedMontoya, Ivan AlonsoBlanco Mesa, Fabio RaulMontoya-Castillo, AndrésBlaszczuk, ArturMoon, Kevin R.Blázquez-Salcedo, Jose LuisMoon, KyuminBLÉ, GAMALIELMora, Jose R.Bloch, Matthieu R.Morabito, Francesco CarloBlok, BorisMoraru, LuminiţaBlutner, ReinhardMorawetz, KlausBocharova, Irina E.Morchid, MohamedBodaghi, MahdiMoreira, CatarinaBodrova, AnnaMoreno, EliasBogachev, Leonid V.Moreno, JaimeBogataj, MarijaMoretti, ValterBogatov, AndreiMoreva, EkaterinaBogolubov, NikolaiMorgado, Welles Antonio MartinezBolonek-Lasoń, KatarzynaMorikawa, MasahiroBolotnov, IgorMorita, SatoruBoltuc, PeterMorosuk, TatianaBonança, Marcus V.S.Moroz, IreneBonanno, ClaudioMorozov, Alexandre V.Bonometto, Silvio A.Morozov, Vitaly A.Bonomo, MatteoMorzfeld, MatthiasBontempi, GianlucaMorzy, MikołajBonyár, AttilaMosquera, MartinBopp, Fritz W.Motahhir, SaadBoragno, CorradoMotsch, SebastienBorgonovi, FaustoMou, JunBorgonovo, EmanueleMoysis, LazarosBormashenko, EdwardMrzljak, VedranBormetti, GiacomoMtz Khramer, DanielBorrego, CarlosMuhammad, NazeerBoschetto, FrancescoMuirhead, StephenBose, RohitMukhamedov, FarrukhBošnjaković, MladenMukherjee, PritamBossé, ÉloiMukhopadhyay, SubhasBossert, MartinMuller, Felipe MartinsBosso, LucianoMuneepeerakul, RachataBosso, PasqualeMunim, Ziaul HaqueBossomaier, TerryMuñoz, Jorge A.Boström, KimMunoz-Pacheco, Jesus M.Bóta, AndrásMunoz-Pacheco, Jesus ManuelBoubchir, LarbiMurari, AndreaBoulama, Kiari GoniMuresan, CristinaBouleux, GuillaumeMurillo-Escobar, Miguel AngelBour, PetrMurino, TeresaBoutet, AntoineMuschik, WolfgangBovino, Fabio AntonioMuthugala, VirajBoyd, Jonathan W.Mylnikov, LeonidBoyvalenkov, PeterMyung-Whun, KimBozorg, MokhtarNaddeo, AdeleBraselton, JamesNaderi, MehdiBratsolis, EmmanuelNagaj, DanielBrauer, DaveNagy, MátéBrault, VincentNaik, Ganesh R.Bravetti, AlessandroNakamura, TadaoBravo Gaete, Moisés FelipeNakano, MarikoBreitsamter, ChristianNakhchi, Mahdi ErfanianBrenner, NaamaNakiboğlu, BarışBretto, AlainNaletina, DoraBrey, JavierNamazi, HamidrezaBrini, FrancescaNambu, YasusadaBrinkman, BradenNami, HosseinBrito, FredericoNapoletano, Brian M.Brkic Bakaric, MarijaNapp, DiegoBroadbridge, PhilipNara, ShigetoshiBrogioli, DorianoNaranjo, LizbethBronkhorst, Curt A.Narayan, Darren A.Broome, MatthewNarayanan, BarathBross, ShragaNarducci, DarioBrumen, BoštjanNastuta, Andrei VasileBruno, Maria FrancescaNataliani, YessicaBruza, Peter D.Nathoo, FaroukBruzzaniti, PaoloNaudts, JanBryndum-Buchholz, AndreaNazari, FoadBrzostowski, KrzysztofNazarkevych, MariyaBudaca, RaduNeagu, Bogdan ConstantinBudini, Adrián A.Nechita, IonBueno, PabloNeda, ZoltanBull, JamesNeed, RyanBulnes, FranciscoNejad, Reza MasoudiBurattini, LauraNejadsattari, FarshadBurlaga, Leonard F.Nelson, KenricBusch, ThomasNenkov, NaydenBuszko, KatarzynaNepomuceno, Erivelton GeraldoButenko, SergiyNeri, IgorButov, LeonidNešumajev, DmitriButtigieg, VictorNeumann, MichaelButusov, DenisNeves Lousada, Sérgio AntónioByeon, HaewonNeves, Leandro A.Caballero-Pintado, Maria VictoriaNeves, Leandro AlvesCacciatori, SergioNghe, PhilippeCacciola, AlbertoNguyen, DinhCaceres, Manuel OsvaldoNguyen, Tan N.Caeiro, FredericoNguyen, Thong TrungCaesarendra, WahyuNi, RanCafagna, DonatoNicholson, DonCafaro, CarloNicolaou, NicolettaCahay, Marc M.Nicolis, OriettaCahoy, DexterNielsen, FrankCai, HaipengNielsen, Jesper KjærCai, NingNielsen, KimCai, ShaoweiNiesen, JitseCai, XiaohaoNiestegge, GerdCaicedo, AlexanderNieuwenhuizen, TheoCaillault, Emilie PoissonNiimi, AyahikoCailliez, FabienNikitenko, AgrisCalcagnile, LucioNikkhah, AminCalderín-Ojeda, EnriqueNikolic, DraganCalegari, RobertaNikulov Aleksey V.Calhoun, JonNin, JordiCaliva, FrancescoNisar, Kottakkaran SooppyCalvo, FlorentNiu, RanCalvo, Hernán L.Nivedita, NiveditaCalvo, HiramNo, AlbertCalvo-Lobo, CésarNobile, Marco S.Calzetta, EstebanNogaret, Alain R.Cámara Chávez, GuillermoNomura, YasuhiroCamati, Patrice A.Nori, FrancoCambria, ErikNoshad, MortezaCampbell, SteveNosonovsky, MichaelCampos Cantón, EricNovikov, Alexander S.Campos Souza, Paulo VitorNowiński, WitoldCandido, RenatoNowosad, JakubCanepa, EdwardNozariasbmarz, AminCang, ZixuanNunes, AbrahamCannon, William R.Nurunnabi, MohammadCanonne, ClémentNyka, KrzysztofCao, BingyangO’Donnell, CianCao, JindeO’Shea, AmyCao, ZehongOaknin, DavidCapasso, AnnaObaiys, SuzanČapek, JanOblak, BlagojeCaponetto, RiccardoOchoa, Guillermo ValenciaCaravelli, FrancescoOdeh, Suhail M.Carballal, AdrianOdriozola, GerardoCarbone, AnnaOehr, PeterCarbone, FrancescoOesting, MarcoCardoso, Katia ReginaOhsawa, YukioCarfì, DavidOizumi, MasafumiCaridad Y Ocerin, Jose MariaOkabe, YutakaCarlo, GabrielOkopinska, AnnaCarmi, AvishyOksiuta, ZbigniewCarotenuto, MarcoOlbrich, EckehardCarpena, PedroOlbryś, JoannaCarrasco, MiguelOldofredi, AndreaCarroll, SeanOlendski, OlegCarvalho, Luiz MaxOlenko, AndriyCarvalho, MónicaOliva, DiegoCasarin, RobertoOliva, PietroCasiraghi, GionaOlivares, FelipeCastaños, OctavioOlive, David J.Castellanos Regalado, Francisco JoséOliveira Panão, Miguel R.Castiglione, ArcangeloOliveira, Marcos Cesar DeCastiglioni, PaoloOliveira, Santiago Del RioCastillo-Soria, Francisco RubénOliveira, Tiago RouxCastro, Luis MauricioOliveri, FrancescoCastro, Paula MaríaOlkhov, VictorCasu, GavinoOllikainen, TuomasCatalano, JacopoOlmos, Pablo M.Caţaron, AngelOng, LawrenceCaticha, NestorOnuki, AkiraCatrini, PietroOohama, YasutadaCattani, CarloOprea, Corneliu IoanCavalcante, Charles CasimiroOravec, JakubCavina, VascoOron, AlexCayco-Gajic, AlexOrtale, RiccardoCazabet, RemyOrtega Sarmiento, SamuelCéleri, Lucas ChibebeOrtigueira, ManuelCelli, MicheleOrtiz De Zárate, José M.Centorrino, SamueleOrtiz-Medina, JosueCeptureanu, Sebastian IonOrus Perez, RaulCercas, FranciscoOsawa, MinoruCerqueti, RoyOsenda, OmarCervantes, JairOsés, Daniel MoratóCervigón, RaquelÓskarsdóttir, MaríaCetto, Ana MariaOsmolovskii, Nikolai P.Ceulemans, ArnoutOsmulski, Pawel A.Chai, RifaiOstrovskii, ValeriiChakrabarti, ArnabOszust, MariuszChallet, DamienOtamendi, Francisco JavierChamberlin, RalphOtmani, AyoubChamkha, AliOtoum, SafaChamoso Santos, PabloOtu, HasanChan, Leo H.Otzen, DanielChan, StephenOurabah, KamelChancellor, NicholasOvalle, María AntoniaChandra, DaryusOverill, RichardChandra, RohitashOverstall, AntonyChang, Lo-BinOvgun, AliChang, VictorOwari, MasakiChang, XiaoOzel, OmurChang, Yao-FengOzilgen, MustafaChantasri, AreeyaOzols, MārisChappell, Thomas M.Paez-Hernández, RicardoCharakopoulos, AvraamPagnini, GianniCharkhkar, HamidPai, Tun-WenCharleston-Villalobos, SoniaPaillard, DidierChatterjee, AtanuPaillusson, FabienChatzidimitriou-Dreismann, C. ArisPait, FelipeChau, Hoi FungPal, Priyo ShankarChechkin, AlekseiPalacios-Laloy, AgustinChechulin, AndreyPalao, José P.Chehri, AbdellahPalazzo, PierfrancescoChemtob, YohannPalczynska, BeataChen, BinqiangPaleologu, ConstantinChen, ChiachungPaliathanasis, AndronikosChen, HangPaliwal, NikhilChen, JincanPałka, PiotrChen, JinjuPalmowski, ZbigniewChen, JunPamucar, DraganChen, JunxinPan, BaoxiangChen, LingenPan, Jeng-ShyangChen, WeiPan, Shaowu (Shawn)Chen, XianzhongPan, YijunChen, YuhuaPandit, RaviChen, YuwenPandya, BhargavCheng, Chi-HoPang, JunCheng, HuanyuPang, XiaodanCheng, Huanyu (Larry)Panzeri, StefanoCheng, MinquanPapana, AngelikiCheng, Szu-ChengPapanastasiou, PanagiotisCheng, YongqiangPapathanasiou, JasonChenu, AureliaPapp, GáborCherifi, ChantalParalič, JánCherniha, Roman M.Paraoanu, GheorgheCherubini, StefaniaParaoanu, Gheorghe SorinChesi, GiovanniPardo Martínez, Clara InésChesneau, ChristophePardo, María Del CarmenChesneau, XavierPark, ChanseokChesnokov, AlexanderPark, Choon-SuChevallier, JulienPark, DonghwiChi, SienPark, SangunChiao, Yu-HsuanParker, JulianChiatti, LeonardoParodi, EmiliaChiba, KazuhisaParr, ThomasChibbaro, SergioPascal, RobertChien, Been-chianPassante, RobertoChih, MingchangPasson, OliverChillali, AbdelhakimPasten, DenisseChing, Tak-ShingPasternak, MateuszChira, CameliaPastore, AdrianoChirikjian, Gregory S.Pastur, LucCho, Jin SeoPastusiak, RadoslawChockalingam, Sriram P.Paszto, VitChoi, JaeyoungPataka, AthanasiaChoi, Moon SubPatel, Ravi G.Choi, YonghoPatikova, ZuzanaChou, RémiPatil, DeepakChou, TungPatil, VikramChoudhary, KrishnaPatriarca, MarcoChoudhury, SayantanPatriarca, RiccardoChow, JamesPatwardhan, AbhijitChudziak, JacekPauk, JolantaCHUI, Kwok TaiPaulevé, LoïcCiaburro, GiuseppePaunković, NikolaCiaglia, Florio M.Pavaloaia, Vasile DanielCiaramelli, ElisaPavlovic, NatasaCiblat, PhilippePavlovich, Pljonkin AntonCiccarello, FrancescoPavlyuk, DmitryCieslak, JakubPavón, DiegoCirovic, GoranPavsič, MatejCisneros-Magaña, RafaelPawela, ŁukaszCiszak, OlafPawłowski, MarcinCiuonzo, DomenicoPazira, HassanCivelli, StellaPederzolli, FedericoClaramunt, ChristophePedraza, JuanCleaver, GeraldPeer, PeterCliff, OliverPei, SenClunie, DavidPei, YanzhongCodetta Raiteri, DanielePejaś, JerzyCofré, RodrigoPejic Bach, MirjanaCohen, AsafPeliti, LucaCohen, GilPelizzola, AlessandroCojocaru, VasilePelster, AxelColangeli, MatteoPeluso, EmmanueleColin, RemyPeña, Francisco J.Colombo, Luigi Pietro MariaPendrill, LeslieColombo-Mendoza, Luis OmarPeng, HaipengColorado-Garrido, DarioPeng, JialiangComesaña, PedroPeng, XindongCometta, LuisPennini, FlaviaConcezzi, MorenoPerarnau-Llobet, MartíConcli, FrancoPereira, SandrinaConstantinescu, Catalin-DanielPereiro, ManuelConstantinou, AnthonyPérez Gutiérrez, MikelContreras-Reyes, Javier E.Pérez Mariño, InésConvertino, MatteoPerez, ArmandoCorreia, JoãoPérez, ElíasCorti, David S.Pérez-Cárdenas, Fernando C.Costa, Gilson A.O.P.Perez-Delgado, Carlos A.Costa, Max Henrique MachadoPérez-Fernández, RaúlCosta, Vítor Antõnio FerreiraPérez-García, VicenteCostabal, Francisco SahliPérez-González, Carlos J.Costache, Romulus-DumitruPérez-Madrid, AgustiCOSTEA, MonicaPerić, ZoranCostin, HaritonPericchi, LuisCotfas, Liviu-AdrianPerkins, WillCouchot, Jean-FrançoisPerlick, VolkerCourbebaisse, GuyPerne, MatijaCousineau, DenisPernice, RiccardoCozot, RemiPerumalla, Kalyan R.S.Craciunescu, TeddyPervez, ZeeshanCraus, MiticaPeskin, UriCrespo Bofill, PedroPeterson, Joseph R.Crippa, PaoloPetkoski, SpaseCristani, MatteoPetroni, FilippoCristian, DumitruPetropoulos, ConstantinosCroccolo, FabrizioPetrovska-Delacrétaz, DijanaCruz, ManuelPetrzela, JiriCruz-Hernández, CésarPetsev, Dimiter N.Csernoch, MariaPetzoldt, AlbrechtCuevas, ErikPfeiffer, MarcoCugliari, JairoPfletschinger, StephanCui, HuijuanPfost, HeinerCunha, Carlo Requião DaPham, Tuan D.Curiac, Daniel-IoanPiacentini, FabrizioCurilef, SergioPiangerelli, MarcoCushman, Samuel APiasecka, MagdaCysarz, DirkPiasecki, KrzysztofCzechowski, ZbigniewPiasini, EugenioD’Abramo, GermanoPidko, EvgenyD’Ammando, FilippoPiepenbrink, KurtD’Angelo, GianniPilař, LadislavDa Luz, Marcos Gomes EleutérioPili, RobertoDabic, MarinaPilipenko, Viacheslav A.Dabija, Dan-CristianPin, PaoloDabirnia, MehdiPineda, MiguelDadlani, AreshPinheiro, FlavioDafonte, CarlosPinho, Armando J.Daher, WajeehPinto, Armando N.Dahmen, DavidPintó, JosepDahyot, RozennPiotrowski, ZbigniewDai, SongsongPipień, MateuszDaivis, PeterPirandola, StefanoDal Maso, FabienPires, Ivan MiguelDaly, KevinPirjan, AlexandruDamaševičius, RobertasPirjol, DanDamián-Ascencio, César EduardoPirogov, SergeyDanaila, LuminitaPisarek, MarcinDaneshazarian, RezaPisarenko, VeronikaDaneshmand, MahmoudPisarev, VasilyDaniel Israeli-Korn, SimonPiskorski, JaroslawDaniels, BryanPistone, GiovanniDapena, AdrianaPitti, AlexDarmon, DavidPizarro, Mario MartínezDarwen, Paul J.Planat, MichelDas, ProdipPlastino, AngelDassios, AngelosPlastino, AngeloDaum, FredPleimling, MichelDavid, SergioPljonkin, AntonDavison, MattPlotnitsky, ArkadyDavydenko, YevhenPogány, Tibor K.Davydovych, Vasyl’Polani, DanielDayoub, IyadPolanski, ArnoldDbouk, TalibPołap, DawidDe Albuquerque Pereira, Wagner CoelhoPolimeno, AntoninoDe Araújo, José CarlosPollard, Blake S.De Bernardi, ElisabettaPollner, PeterDe Bragança Pereira, Carlos AlbertoPomares, SaúlDe Brevern, Alexandre G.Pombo, NunoDe Decker, YannickPonomaryov, VolodymyrDe Diego Onsurbe, José AntonioPons, MichelDe Faria, Sérgio M.M.Pop, NicolaeDe Gennaro, LuigiPopa, Gabriel NicolaeDe Jesus, José FernandoPopa-Wagner, AurelDe La Fraga, Luis G.Popescu, Theodor D.De La Pena, Lisandro HernandezPopkov, Yuri S.De Laborderie, JérômePorta, AlbertoDe Menezes, Marcio ArgolloPortillo, DavidDe Meo, PasqualePortugali, JuvalDe Mingo López, Luis FernandoPorzio, AlbertoDe Oliveira Junior, SilvioPosada-Quintero, Hugo F.De Oliveira, Mário JoséPósfai, MártonDe Pasquale, AntonellaPoti, ValerioDe Saá Guerra, YvesPouliaris, ChristosDe Sanctis, Angela AnnaPoulkov, VladimirDe Vos, AlexisPourpanah, FarhadDe Wolf, ErickPowell, Robert J.De, AmritPrakash, ChetanDeaconu, Sorin IoanPrasad, MukeshDeamer, DavidPrati, RonaldoDebono, Carl JamesPratt, ScottDębowski, ŁukaszPreda, VasileDecu (Marinescu), SimonaPręgowska, AgnieszkaDecu, SimonaPrentner, RobertDedu, SilviaPrice, HuwDeffner, SebastianPrisecaru, TudorDeFord, DarylProesmans, KarelDegollado, Juan CarlosProkopenko, MikhailDeguchi, KengoPross, AddyDehdashti, ShahramProtopapadakis, EftychiosDehesa, Jesu’s S.Prudhomme, SergeDeinum, Eva E.Prus, BarbaraDeiters, UlrichPrusak, Błażejdel Campo, AdolfoPrykarpatsky, AnatoliyDel Rio Oliveira, SantiagoPrzybyłek, MaciejDel Val, LaraPsannis, KonstantinosDelcea, CameliaPuebla, RicardoDeLellis, PietroPuechmorel, StephaneDelgado, FranciscoPuerta, José MiguelDelrieux, ClaudioPuerto, Inés M. DelDemetrius, LloydPuglisi, AndreaDemirel, YasarPujol, Francisco A.Deng, DianliangPyshkin, PavloDeng, HongzhongQazi, AbroonDeng, XiaofeiQi, ChaoDeng, YanxiangQi, Hou-DuoDeng, YongQian, HongDeniziak, StanisławQian, XiaofengDepolli, MatjazQiao, JunweiDeppman, AirtonQiao, TongDequen, GillesQin, SanboDevorshak, ChristinaQiu, DaowenDhimish, MahmoudQuapp, WolfgangDi Bona, GianpaoloQuattrone, AldoDi Cosmo, FabioQueirolo, ElenaDi Crescenzo, AntonioQueiros, Silvio M. DuarteDi Liberto, GiovanniQueluz, Maria PaulaDi Martino, FerdinandoQuessy, Jean-FrançoisDi Matteo, LucioQuoreshi, A.M.M. ShahiduzzamanDi Pasquale, NicodemoQyyum, Muhammad AbdulDialynas, KonstantinosRadhakrishnan, ChandrashekarDiana, GiovanniRadic, DankoDias, Sofia BalulaRadicchi, FilippoDiaw, AbdourahmaneRadicic, DraganaDíaz Cardell, SaraRadivilova, TamaraDickins, TomRadu, FlorinDieks, DennisRadziemska, MajaDiez, YagoRafał, Marcin LaskowskiDill, KenRagulskis, MinvydasDing, JintaiRahimi Tabar, Mohammad RezaDing, YuanRahimian, ArdavanDionísio, AndreiaRahimi-Gorji, MohammadDitl, PavelRaitoharju, JenniDjebedjian, BergeRajamani, KumarDjenouri, YoucefRak, RafałDo, Dinh-ThuanRakowski, AndrzejDo, YounghaeRamdin, MahinderDobre, Robert-AlexandruRamírez Pacheco, Julio CésarDodonov, ViktorRamirez, Pedro E.Dokuchaev, VyacheslavRamirez, RafaelDolega, BoguslawRamirez-Pastor, Jose AntonioDöller, MarioRamirez-Reyes, AbdielDomenici, ValentinaRamirez-Rojas, AlejandroDomínguez, Luis VergaraRamos, PedroDomínguez-Morales, ManuelRamos, RaulDong, BoxiangRampino, SergioDong, LichunRamstead, Maxwell J.D.Dong, YunquanRana, SwapanDorato, MauroRangarajan, AnandDos Santos, RicardoRangel, VictorDouglas, JackRangel-Hernandez, Victor HugoDoulamis, AnastasiosRanjan, ReeteshDoulamis, NikolaosRantalainen, MattiasDouligeris, ChristosRao, Suhasini SubbaDowning, CharlesRasche, ChristophDoyon, NicolasRashad, Ahmed M.Drąg, PawełRashid, MuhammadDragomir, AlinRashidi, JouanDreißigacker, VolkerRassia, Michael (Michail) Th.Drezewski, RafalRastelli, GianlucaDrikakis, DimitrisRáth, BalázsDrissi-Habti, MonssefRau, A. Ravi P.Drozdz, StanislawRaudino, AntonioDrugowitsch, JanRauh, AlexanderDrutarovsky, MilosRavelet, FlorentDu, JiulinRavier, PhilippeDu, YunchengRay, ChristianDuarte, Anderson RibeiroRaymer, Michael L.Duch, WłodzisławRazeghi, BehroozDudin, AlexanderReboiro, MartaDulău, MirceaRecamier, JoséDuque, OscarReddy, Yenumula B.Dutta, AtriReeke, George N.Dutton, ZacharyReeves, GalenDux-Santoy Hurtado, LydiaRegmi, SamundraDytso, AlexRehman, Junaid UrDzemyda, GintautasReich, SebastianE., JiaqiangReimhult, ErikEchchabi, AbdelghaniReinhart, Wesley F.Ecke, ThorstenReis, António HeitorEckold, GötzRempel, Erico LuizEdge, Michael D.Ren, QiangEduardo Espitia, HelbertRenaud, ChristopheEgli, RamonRenedo, Carlos J.Egolf, Peter W.Renevey, PhilippeEgorov, VladimirRengarajan, AmirtharajanEhresmann, AndreeRenza, DiegoEid, Mohamed RabeaResende, PedroEisencraft, MarcioResnina, NataliaEl Assad, SafwanRetscher, GüntherEl Euch, HichemRevnic, CorneliaEl Moumni, HasanRey, DavidElena, LucchiReyad, OmarEleuch, HichemReyes Belmonte, Miguel ÁngelEliwa, MohammedReynaud, RogerEl-Khazali, ReyadRezapour Mashhadi, Mohammad MahdiEllahi, RahmatRezk, AhmedElliott, ThomasRhoades, DavidElmogy, MohammedRiani, MarcoEl-Nabulsi, Rami AhmadRiba Ruiz, Jordi-RogerElouard, CyrilRibba, AntonioElsadany, Abdelalim A.Ribeiro Gabriel, Paulo HenriqueElze, ThomasRibeiro, FabianoEmeršič, ŽigaRibeiro, Haroldo V.Endres, DominikRibeiro, Haroldo ValentinEngdahl, JohanRibeiro, PatrickEramo, VincenzoRibeiro, RitaErguvan, MustafaRibeiro, TiagoErnst, GernotRibeiro, VitorErrera, Marcelo R.Richert, RankoErukhimov, VictorRichman, JoshuaEryilmaz, AtillaRichmond, PeterEscamilla Herrera, Lenin FranciscoRichter, HendrikEscamilla-Rivera, CeliaRichter, Wolf-DieterEscobar-Jiménez, RicardoRidder, AdEscolano Ruiz, FranciscoRiechers, Paul M.Escudero, JavierRigby, DavidEsfeld, Michael-AndreasRighi, RodrigoEsparcia, JavierRighi, SimoneEsposito, GianlucaRiguidel, MichelEsquivel, Rodolfo O.Riis, SorenEtesi, GaborRioul, OlivierEtesse, JeanRivas, ÁngelEun, Hee-ChangRivero, Cristian RodriguezEvans, MichaelRivolta, Massimo WalterEvans, Peter W.Rizal, ConradEzquerra, TiberioRizman Zalik, KristaFabregas, Albert Guillen I.Robert, TristanFabrikant, IlyaRobertson, DavidFabris, JúlioRocca, Mario C.Faes, LucaRocha, J. LeonelFaggini, MarisaRocha, Luiz Alberto OliveiraFahs, MarwanRoco, José Miguel MateosFaísca, PatríciaRodger, JamesFalceto, FernandoRodriguez Ariza, LazaroFalco, Pasquale DeRodriguez Diaz, Camilo ArturoFaller, HuguesRodríguez Fonollosa, JavierFalquet, GillesRodríguez Martín, José AntonioFan, Deng-PingRodríguez, FranciscoFan, YuruiRodriguez, Miguel A.Fang, RuixianRodriguez, NibaldoFarah, Mohamed Amine BenRodríguez-Baena, Domingo SavioFarahi, AryaRodríguez-Lara, Blas ManuelFarantos, Stavros C.Roduner, EmilFarmer, MichaelRoelofs, LukeFarnsworth, Keith D.Rofouie, PardisFasano, GiovanniRoga, WojciechFaseeh Qureshi, Nawab MuhammadRogolino, PatriziaFasquelle, ThomasRogovoy, Anatoly A.Fassas, Athanasios P.Roldán Gómez, Juan JesúsFatkullin, IbrahimRomano, VittorioFaundez-Zanuy, MarcosRomeo, LucaFavretti, MarcoRomero, RicFazakas, EvaRomero-Rochin, VictorFebres, GerardoRomo Fernández, Luz MaríaFedorov, AlexeyRondoni, LambertoFeinstein, ZacharyRonnow, DanielFelice, DomenicoRopero, JorgeFeng, JunRopp, ChadFeng, RuiRosas, FernandoFeng, YunlongRosas-Ortiz, OscarFennell, ChristopherRosenbluh, MichaelFernández Bariviera, AurelioRossetti, GiulioFernández Fernández, Francisco JavierRossi, GiancarloFernández Vázquez, EstebanRossi, Julio DanielFernandez, AlbertoRossi, MassimilianoFernández-Fontelo, AmandaRossini, DavideFernández-Luqueño, FabiánRosu, HaretFernandez-Veiga, ManuelRota, RiccardoFernando, GarcíaRoth, Peter M.Ferracuti, FrancescoRothkrantz, LeonFerraro, DarioRotundo, GiuliaFerraro, ElenaRoudi, YasserFerreira, ArturRouttenberg, TirzaFidaleo, FrancescoRovelli, CarloFields, ChrisRoy, ArkoFietkiewicz, Kaja J.Roy, DebrajFigaj, RafałRozas-Fernandez, AlbertoFilchenkov, AndreyRozinajova, VieraFilho, Mauro FazionRubio Alonso, HiginioFilippov, AnatolyRubio, F. JavierFill, Hans-GeorgRudnicki, ŁukaszFiore, UgoRudžionis, VytautasFirpo, Marie ChristineRuiz, MarianoFischer, DanielRukavina, SanjaFistul, MikhailRullière, DidierFiumara, GiacomoRulseh, Aaron MichaelFlamini, FulvioRungi, ArmandoFloreani, JosancoRuocco, GiancarloFlores Tapia, DanielRuppeiner, GeorgeFlores, J.C.Rusu, ValentinaFlórez-Orrego, DanielRyabov, ArtemFlorio, GiuseppeRydzewski, JakubFloros, GeorgeRyu, JungheeFocardi, SergioRyzhii, Maxim V.Focardi, StefanoSaadane, RachidFodor, Petru S.Saarela, MirkaFogarty, ThomásSabet Jahromi, Mohammad NaserFomin, AlexeySabin, CarlosFonollosa, Javier RodríguezSabín, CarlosFonseca-Luengo, DavidSadatnejad, KhadijehFont Clos, FrancescSadeghinezhad, EmadForchhammer, SørenSadek, Rowayda A.Forest, GregSadjadi, Firooz A.Formato, AndreaSaeedian, MeghdadFormentin, MarcoSafaai, HoumanFormicola, ValerioSafaei, Mohammad RezaForti, MauroSafdari Shadloo, MostafaFortuna, LuigiSafron, AdamFrämling, KarySagar, Robin P.Franchino-Viñas, SebastiánSaha, AshirbaniFrancisco, Gómez Aguilar JoséSaha, SubrataFranco, Admilson T.Sahin, BekirFranco, AlessandroSahu, SubinFränti, PasiSaia, RobertoFrascolla, ValerioSaïda, Ahmed BenFraternali, FernandoSaika-Voivod, IvanFredianelli, LucaSakaguchi, HidetsuguFreedman, KevinSakalauskas, EligijusFresch, BarbaraSałabun, WojciechFrid, VladimirSalamon, PeterFriederich, SimonSalazar, AddissonFriedrich, Christoph M.Salazar-Pereyra, MartinFriston, KarlSales, Rita De Cássia MendonçaFrixione, MarcelloSalgado, RafaelFrontoni, EmanueleSalimi, MehdiFrydryszak, AndrzejSalinet, JoaoFu, ChaoSalnikov, VladimirFu, ChenguangSalomon, PeterFuentes, Miguel A.Salvador, Marisela RodríguezFujieda, TadashiSalvetr, PavelFujigaki, MotoharuSamengo, InésFujiwara, TakaoSampson, AaronFÜLÖP, BAZSÓSamsten, IsakFurmańczyk, KonradSanchez Granero, Miguel AngelFuruta, HiroshiSanchez Lasheras, FernandoGabriele, MagnaSánchez López, FernandoGabuzda, DeniseSanchez, DavidGadomski, AdamSanchez, Mauricio A.Gagnadoux, FredericSánchez, René-VinicioGajic, DragoljubSánchez-Cañizares, JavierGal, ZoltánSanchez-Oro Calvo, JesusGalam, SergeSanchez-Soto, LuisGalán, José ManuelSánchez-Torres, Juan DiegoGalhano, AlexandraSANCIBRIAN, RAMONGalicia-Luna, Luis A.Sandev, TrifceGalilea, Adrian RamirezSandoval, LeonidasGallavotti, GiovanniSands, DavidGallegos-Funes, FranciscoSang, Tzu-HsienGallerati, AntonioSanislav, TeodoraGalli, Brian J.Sanjuán, Miguel A.F.Gallucci, FrancescoSantiago, JJesus DeGammaitoni, LucaSantiago, Pablo MesejoGan, MinSantolini, MarcGangardt, Dimitri M.Santos, AndrésGangsei, Lars ErikSantos, Fernando P.Ganguly, DebasisSantos, José IgnacioGannon, Anthony J.Santos, Marcelo FrancaGao, FeiSantucci, ValentinoGao, PeichaoSanz Ortiz, Ángel SantiagoGao, ShimingSapena, JuanGao, WeiSara, RadimGao, XiangyunSaracevic, Muzafer H.García March, Miguel ÁngelSarath, BharatGarcia Pérez, GuillermoSarhan, AmanyGarcía Salcedo, RicardoSarkar, Nurul I.García, Jesus EnriqueSarker, SubrataGarcía, Manuel PérezSarlis, NicholasGarcía, Miguel TorresSarracino, AlessandroGarcía-Alcaraz, Jorge LuisSaslow, WayneGarcia-Beltran, Carlos D.Sason, IgalGarcía-García, ReinaldoSassi, SadokGarcia-Hernandez, Jose JuanSassoli De Bianchi, MassimilianoGarcía-Morales, VladimirSathar, E. I. AbdulGarcía-Ordás, María TeresaSavoiu, GheorgheGarcia-Perez, ArturoSawerwain, MarekGarcía-Sánchez, José RafaelSayed, Wafaa S.Garcin, MatthieuSbert, MateuGardner, AndyScalera, LorenzoGargano, FrancescoScaramozzino, PasqualeGargiulo, Gaetano D.Scardapane, SimoneGarnier, Nicolas B.Scarpino, Samuel V.Garrido, MarioScatá, MarialisaGärttner, MartinSchaefer, BenjaminGartus, AndreasSchaeffer, Satu ElisaGarza, JorgeScheeren, EduardoGąska, AdamSchifanella, ClaudioGatsonis, ConstantineSchlitter, JürgenGattone, Stefano AntonioSchmandt, BastianGavet, YannSchmidt, Eric A.Gavrilov, MomčiloSchmidt, HelmutGavrilut, Alina CristianaSchmidt, Jan H.Gavrovska, AnaSchmitz, GunnarGay-Balmaz, FrancoisSchnoerr, DavidGazeau, Jean-PierreSchoen, ChristianGe, HaoSchoffelen, Jan-MathijsGega, JerzyScholkmann, FelixGehring, TobiasScholz, MichaelGeiselhart, MarvinSchormans, JohnGenç, Ali İhsanSchossau, JoryGençağa, DenizSchrefler, BernhardGenovese, AngeloSchreiber, AndreasGeorge, FlorenceSchreiber, IgorGeorge, StanescuSchulman, LawrenceGeorgieva, TsvetelinaSchütze, OliverGerbens-Leenes, P.W.Schwartzman, ArminGerman-Sallo, ZoltanSchweitzer, FrankGerogiannis, Vassilis C.Schwenker, FriedhelmGhany, Kareem Kamal A.Schwonnek, RenéGhasemi-Gol, MajidSciaraffa, NicolinaGheorghe, DumitrascuSciubba, EnricoGheorghe, GrigorasSedakov, ArtemGheorghe, MarianSedighi, TabassomGherghina, Ştefan CristianSedmidubský, JanGhim, Cheol-MinSegall, RichardGhosh, IndranilSegreto, LucianoGhosh, SubirSeljan, SanjaGhosh, SucharitaSellitto, AntonioGhosh, Sujit KumarSemenkin, EugeneGiacometti, AchilleSemenov, SemenGiannakis, KonstantinosSemeraro, OnofrioGiannone, FrancescaSemin, BenoitGiannoulakis, StamatiosSemkov, KrumGibbs, David L.Senatore, RosaGil, DavidSene, NdolaneGill, Richard D.Seo, HwajeongGil-Villegas, AlejandroSeo, WonchulGiordano, StefanoSepas-Moghaddam, AlirezaGiorgi, Gian LucaSerackis, ArturasGiovagnoli, RaffaelaSergioli, GiuseppeGiráldez Crú, JesúsSerna Barquera, Sergio AlonsoGiraldo, BeatrizSerota, RostislavGiuliani, AlessandroSerrien, BenGiven-Wilson, ThomasServedio, Vito D.P.Glavatskiy, KirillSetuha, Alexey V.Gligorijevic, DjordjeShafiq, OmairGlotsos, DimitrisShahandeh, FaridGlowacz, AdamShahriar, Md RifatGnatowski, AndrzejShahverdiev, ElmanGnatyuk, SergiyShahzad, Muhammad WakilGoh Oon Soo, JoshuaShakhovska, NataliyaGohari, Amin (Aminzadeh)Shalyt-Margolin, AlexanderGolovnin, Oleg K.Shamshirband, ShahabGomes-Silva, FrankShang, XiaopuGómez Aguilar, José FranciscoShang, YilunGomez Extremera, ManuelShao, DangdangGomez, HectorShapovalov, AlexanderGómez, VicençSharp, Kim A.Gómez-Aguilar, José FranciscoSharpe, Shaun M.Gomonay, OlenaShaw, HarryGonçalves, Maria EsmeraldaShaw, Timothy I.Gong, HaijunShayevitz, OferGong, JiaweiShcherbakov, PavelGong, XinqiShcherbakov, RobertGontis, VygintasShe, KunGonzález De La Rosa, Juan-JoséShe, QiGonzález, Carlos M. MoraSheehan, DanielGonzalez-Ayala, JulianShekaramiz, MohammadGonzález-Gaxiola, OswaldoShen, BoGonzález-López, AntonioShen, TongyeGonzalez-Lopez, Veronica AndreaSheng, Yu-BoGorban, AlexanderShepelyansky, Dima L.Gorban, Alexander N.Sheppard, Charles JohannesGórnicki, KrzysztofSheremet, MikhailGorodetsky, AlexShestakov, Nikolay. V.Gosak, MarkoShevchenko, Vladimir Ya.Gotsis, KonstantinosShevchuk, Igor V.Grabowska, KarolinaShevgunov, TimofeyGradeck, MichelShi, FENG BILLGradojevic, NikolaShi, JianGrafen, AlanShih, DavidGraffelman, JanShihavuddin, ASMGrahovac, DanijelShikano, YutakaGrandhi, SrimannarayanaShin, Won-YongGrangier, PhilippeShiner, JohnGraovac, JelenaShirowzhan, SaraGrassberger, PeterShkliarevsky, GennadyGrattieri, MatteoShrapnel, SallyGraziani, Frank R.Shtub, AvrahamGrebenkov, Denis S.Shu, HaiGrechuk, BogdanShum, KennethGreco, SalvatoreShushi, TomerGregorio, Alessandro DeSiami-Namini, SimaGregorio, Paolo DeSibani, PaoloGreiner, DavidSicilia-Montalvo, Juan AntonioGrendar, MarianSiddique, JavedGrigolini, PaoloSignorini, Maria G.Grigoriu, Mircea D.Sillanpää, MikaGrill, RomanSilva, AdãoGrilli, LucaSilva, Filipi NascimentoGrima, RamonSilva, IvanovitchGrisolia, GiuliaSilva, Jorge F.Grmela, MiroslavSilva, Luiz Eduardo VirgilioGroot, Oliver DeSilva, Luiz Eduardo Virgilio DaGruber, Marvin H.J.Silva, Maisa MendonçaGu, XianmingSilva, RuiGuarnieri Calò Carducci, CarloSim, Heung-SunGubar, ElenaSim, HyogiGudder, StanleySimić, SrboljubGuemez, JulioSimões, NádiaGuendelman, EduardoSimon, JoanGuergachi, AzizSimonucci, StefanoGuerreiro, Gracinda R.Simurda, DavidGuerrero, YolandaSindi, SuzanneGui, NanSing, Swee LeongGui, WenhaoSinger, GonenGuidotti, PatrickSingh, RahulGuijarro, FranciscoSingh, SatyaGuillén, AlbertoSiqueira, Hugo ValadaresGuindani, MicheleSirigina, Rajendra PrasadGulasekaran, RajaguruSivasubramanian, JayaharGuney, DurduSiwik, LeszekGünlü, OnurSkilling, JohnGünther, UweSkinderowicz, RafałGuo, HongSkoglund, MikaelGuo, Jiun-InSkordas, EfthimiosGuo, YuzhuŠkrinjarić, TihanaGuo, Zeng-YuanSładkowski, JanGurvitz, ShmuelSlager, Robert-JanGutub, AdnanSlemrod, MarshallGuzik, AgnieszkaSloutskin, EliGuzmán, EduardoSmall, MichaelGuzmán, LevŚmieja, MarekHa, Jong MoonSmirnov, SemyonHachaj, TomaszSmith, Michael A.Hadar, OferSmolyanitsky, AlexHaddoud, Mohamed YacineSmotrovs, JurisHaeusler, EdwardSnider, JosephHaider, FasihSobh, NahilHalamek, JosefSofos, FilliposHaller, AlinaSohn, InsooHalliday, DavidSokolov, OleksandrHalvarsson, DanielSokolovskiy, Mikhail A.Hamada, MohamedSolin, NiclasHamamura, MasanoriSoloviev, Vladimir NikolayevichHameroff, StuartSoman, RohanHamm, RobertSong, DongHammad, FayçalSong, DongjinHamori, ShigeyukiSong, QifanHamza, BenSong, Seok-ZunHan, PengSong, SeunghoanHandley, WillSong, XueyuHanias, MichaelSonnino, GiorgioHanratty, TimothySoon, WillieHansen, Lee D.Sørensen, ØysteinHao, JiaaoSørensen, Preben GraaeHarremoës, PeterSorrentino, AntonioHartmann, CarstenSossa, HumbertoHashemkhani Zolfani, SarfarazSousa, Ana Margarida Luís DeHashim, KhalidSousa, Joao MiguelHasimoto-Beltran, RogelioSousa, XulioHassaballah, M.Souza, Jeferson AvilaHassouni, AbdelhakSouza, MaxHatemi-J, AbdulnasserSpagnolo, BernardoHatzivasilis, GeorgeSpagnolo, NicolòHaven, EmmanuelSparavigna, Amelia CarolinaHavens, TimothySpencer, EdmundHavis, NeilSperandio, MauricioHawk, JeffreySperlì, GiancarloHawk, Jeffrey A.Sponar, StephanHayashi, MasahitoSpooren, JanHe, FeiSreckovic, Vladimir A.He, JiangenSrikanth, R.He, JiayuanSrivastava, MadhurHe, JiguangSrivastava, VishalHe, WeifengStables, RyanHe, XiaohaiStacchezzini, RiccardoHe, YurongStaniec, KamilHealey, RichardStanislavsky, AleksanderHeberger, KarolyStanley, R. JoeHedayatifar, LeilaStannarius, RalfHeffernan, EmmaStarosolski, RomanHegedűs, FerencStaudte, RobertHeidari, MaziarStawarz, MarcinHeidar-Zadeh, FarnazStecklina, OliverHeideman, WarrenStefanatos, DionisisHeinonen, MarkusStefańczyk, MaciejHeise, Herbert MichaelStefanou, PavlosHejna, BohdanStefanov, AndrejHenchman, RichardStefanova, KremenaHenriksson, JohanSteinbrener, JanHerisanu, NicolaeStella, LorenzoHerman, MichaelStendardo, StefanoHernández, AlfredoStentoft, LarsHernandez, InmaStephanovich, VladimirHernandez, Oliver GutierrezŠter, BrankoHernández-Fernández, AntoniStević, ŽeljkoHernández-Pérez, IvánStewart, MarkHerrera Corral, Gerardo A.Stinis, PanosHerrmann-Pillath, CarstenStipčević, MarioHess, KarlStodola, PetrHewings, GeoffreyStoica, Ovidiu CristinelHicks, YuliaStone, EmilyHidalgo, ArturoStoneking, MatthewHigashikawa, KoheiStoof, Henk T.C.Higgs, PaulStoyanov, BorislavHigson, EdwardStramaglia, SebastianoHill, Peter AlecStreimikiene, DaliaHillery, MarkStruchtrup, HenningHilliard, JitkaStrutz, TiloHinde, ChrisStruzik, Zbigniew R.Hino, HideitsuStrzałka, DominikHirose, KeiStübinger, JohannesHirsch, MatíasStuckey, William M.Ho, NhatStudenka, BreannaHoang, Hong-MinhŠtys, DaliborHoecker-Martinez, MartinSubasi, AbdulhamitHoel, ErikŠubelj, LovroHoffmann, Karl HeinzSubramanian, KarthikeyanHofmann, RalfSuciu, AlinHolik, FedericoSugasawa, ShonosukeHolmes, JeremySugimoto, KounHolovatch, YurijSuh, Jin-YooHolst, AndersSuh, Young-KyoonHolubec, ViktorSukharev, SergeiHong, Song-NamSukin, IvanHorák, JakubSulis, WilliamHorel, EnguerrandSułowicz, MaciejHorne, Rosemary S.C.Sun, GuanghaoHorvát, SzabolcsSun, Guo-HuaHorváth, MiklósSun, HuaHorwitz, LawrenceSun, HuafeiHorwitz, Lawrence P.Sun, Jia RuiHosseinian-Far, AminSun, KeHosseinidehaj, NedasadatSun, Li-HsienHosseinzadeh, MehdiSun, YaoHotza, DachamirSundar, V.S.Hou, JieSunkara, VikramHron, KarelSusa, CristianHsin, Kun-YiSutherland, Roderick I.Hsu, Li-YiSvozil, KarlHu, ChangweiSyed, Mujahid N.Hu, FeiSyriopoulos, KonstantinosHu, JingtianSzabó, CsabaHu, MaocongSzabo, GyorgyHu, RuifengSzczypiorski, KrzysztofHua, ZhongyunSzederkényi, GáborHuang, ChimingSziebig, GaborHuang, E-WenSzkutnik, WłodzimierzHuang, FamingSzopa, MarekHuang, GuangweiSzyc, ŁukaszHuang, Guan-ShiengTabrikian, JosephHuang, HantaoTadokoro, YukihiroHuang, HanwenTaha-Tijerina, José JaimeHuang, Jin-FengTajbakhsh, Shahriar EtemadiHuang, PengdaTakai, KazuyukiHuang, Shou-Hsuan StephenTakeuchi, YukiHuang, TaoTakeya, SatoshiHuang, WenTalbert, DougHuang, XianzhengTaler, DawidHuang, YongxiangTama, Bayu AdhiHuber, MarcoTamascelli, DarioHuber, Roland G.Tamblyn, IsaacHuerta Cuellar, GuillermoTan, VincentHuet, SylvieTan, ZhiyuanHughes, JamesTanaka, TakashiHuh, JoonsukTaneco-Hernández, Marco AntonioHung, FranciscoTang, LongtengHung, Jui-ChengTang, WeiHussain Kazim, AliTang, YongchuanHussain, ZahirTangaro, SoniaHussein, MostafaTaniguchi, ShigeruHussein, MoustafaTanimoto, JunHutchcroft, TomTao, JianjunHutter, MarcusTapias, DiegoHwang, GraceTarasov, Vasily E.Hwang, Yin-TsungTauber, Uwe C.Hyvärinen, AnttiTaufer, EmanueleIana, Vasile-GabrielTavanti, FrancescoIannace, GinoTebaldi, ClaudioIasiello, MarcelloTehranipoor, SaraIbanez-Molina, Antonio J.Teixeira, João C.A.Ibañez-Molina, Antonio JoséTelesca, LucianoIbrahim, Mohamed Nabil FathyTenreiro Machado, José A.Idrisov, EdvinTenreiro, ClaudioIeracitano, CosimoTeo, How Wei BenjaminIglesias, José RobertoTeomy, EialIglói, FerencTermini, DonatellaIgreja, JoséTessarotto, MassimoIiyama, YutaroTettamanzi, GiuseppeIjaz, Muhammad FazalTetteh, GilesIkegami, TakashiTeuerle, MarekIkehara, KenjiThebault, KarimIleanu, Bogdan-VasileThiagarajan, Jayaraman J.Iliescu, TraianThompson, GerardIliyasu, AbdullahThompson, JeffreyImran, MuhammadTian, LiyunIngebrigtsen, TrondTian, ZehuaInglada-Pérez, LucíaTibullo, VincenzoInsinga, Andrea RobertoTikhonenko, OlegIoan-Lucian, PopaTimme, NicholasIovino, VincenzoTimofeyev, IlyaIrfan, MuhammadTimpanaro, Andre M.Isaak, GarthTino, Guglielmo MariaIsaic-Maniu, AlexandruTinoco, Hector A.Isar, AurelianTipeev, AzatIshii, AkiraTishchenko, VictorIshikawa, AtushiTiwary, PratyushIsidro San Juan, José MaríaTkach, MykolaIslam, ShamaTlelo-Cuautle, EstebanIsmail, AdielTlili, IskanderIsmail, MuhammadTobler, RayIsmail, Samar M.Todorov, ValentinISOBE, MasaharuTodorova, NevenaIsomura, TakuyaToffano, ZenoIsvoranu, DragosToker, DanielIvanov, DenisToma, AidaIvanov, PlamenTomasevic, IgorIzonin, IvanTondi, BenedettaIzutsu, JunTopp, KairstyIzydorczyk, JacekToral, RaúlJabrane, YounesToranzo, Irene V.Jachymski, KrzysztofToroš, MarkoJafar, Syed A.Torp, OlavJaferzadeh, KeyvanTorregrosa, Juan R.Jagodzinski, FilipTorres Del Castillo, Gerardo F.Jahromi, RezaTorres, AbelJajcay, NikolaTorres, Claudio E.Jajuga, KrzysztofTorres-Arenas, JoséJakóbczyk, LechTorri, Marco Danilo ClaudioJamali, Seyed HosseinToscano, FabricioJameel, ShoaibToscano, Fernando SolerJamoos, AliTosic, PredragJamshidi, PooyanToups, Zachary O.Janetzko, DietmarTramelli, AnnaJang, Han SeungTramontana, FabioJang, JinwooTran, DatJang, Ju WookTravieso-González, CarlosJankovic, RadmiloTrejo, LuisJanßen, GisbertTrias, F. XavierJanssens, EwaldTribelsky, Michael I.Janus, JakubTrifina, LucianJasielec, Jerzy J.Trifonov, PeterJasiński, KrzysztofTrifonov, Peter V.Jasiński, MarekTrofimov, EvgenyJaubert, Jean-NoëlTrujillo, Logan T.Jauregui, Juan CarlosTrybek, PaulinaJauregui, MaxTsai, Che-WeiJaúregui, RocíoTsallis, ConstantinoJáuregui-Vázquez, DanielTsaramirsis, GeorgiosJaved, FarrukhTsekov, RoumenJaworski, MaciejTseng, Chih-yuanJennings, Robert C.Tsimpiris, AlkiviadisJeon, GwanggilTsinos, Christos G.Jeon, Jun-CheolTsionas, MikeJeong, KabgyunTsukahara, TakahiroJeong, SeongahTsuneda, AkioJessa, MieczyslawTull, SeanJheeta, SohanTumulka, RoderichJhwueng, Dwueng-ChwuanTung, Chih-KuanJi, Un CigTura, JordiJiang, JunleTurgeon, MaximeJiang, LiangxiaoTurkheimer, Federico EdoardoJiménez-Barbero, JesusTusset, Angelo MarceloJin, Bih-YawTutzauer, FrankJin, FengpingTveit, Tor-MartinJing, DaleiTyrcha, JoannaJinwoo, LeeTyukin, IvanJo, JunghyoTyutyunov, YuriJohansen, SorenUchiyama, YusukeJohnson, Don H.Ueltzhoeffer, KaiJohnson, JeffUl Haq, RizwanJohnson, KippUlanowicz, RobertJonah, OlusegunUllah, AminJones, ChrisUlloa, CarlosJones, Karl O.Ungar, AbrahamJordan, AndrewUngerfeld, Emilio M.JORDÁN, FerencUnruh, DominiqueJou, DavidUricchio, LawrenceJouini, NoureddineUsai, ElioJovanoski, ZlatkoUshakov, Sergey V.Jull, Anthony John T.Utev, SergeyJun, Byung-MoonUwe, FranzJun, YonggunVaezi, MojtabaJung, HaejoonVaiciukynas, EvaldasJung, JasonValdes-Hernandez, AndreaJung, KurtValdivia, Juan AlejandroJung, Young-DaeValencia, Jose FernandoJuretić, DavorValencia-Cárdenas, MarisolJusup, MarkoValencia-Garcia, RafaelJuszczuk, PrzemysławValencia-Palomo, GuillermoK. Imlau, MircoValero-Mas, Jose J.Kaabouch, NaimaValiente, DavidKabir, AshadVallianatos, FilipposKaczmarzyk, JanVallicelli, Elia ArturoKadry, SeifedineValls, AidaKagan, LonidValsesia, DiegoKahl, GerhardValtierra-Rodriguez, MartinKai, Ji-JungValverde-Albacete, Francisco J.Kalachev, AlexeyVan Albada, SachaKalenkova, AnnaVan Den Ende, JefKalita, PiotrVan Der Zwaard, StephanKallen, VictorVan Lith, JannekeKalliopi, TrohidouVán, PéterKalogeropoulos, NikolaosVance, EricKamola, MariuszVancea, Ion VasileKanai, RyotaVander Linden, MarcKanbur, Barış BurakVangala, HarishKang, BingyiVarga, Lajos KárolyKang, Dae-KiVargas, BorjaKang, Dong HyunVariny, MiroslavKang, ZihoVarotsos, PanayiotisKania, KrzysztofVarotsos, Panayiotis A.Kanniainen, JuhoVarshney, Lav R.Kanó, Izabella SzakálnéVasconcellos, Klaus L.P.Kao, ChihwaVashisth, HarishKapitaniak, TomaszVasilakos, Athanasios V.Kaplan, Ilya G.Vasko, Ivan Y.Kaplan, RaphaelVassel, SergeiKaplun, DmitriiVatalaro, FrancescoKappes, MartinVaughan, JosephineKapuscinski, TomaszVavoulis, Dimitrios V.Karabasevic, DarjanVázquez Hurtado, FedericoKaragrigoriou, AlexVázquez Polo, Francisco JoséKarakasidis, TheodorosVazquez-Araujo, FranciscoKarakatič, SašoVazquez-Leal, HectorKaramitsos, SotiriosVazquez-Vilar, GonzaloKarawia, Abdel Rahman A.Vecer, JanKarayiannis, NikosVega-Oliveros, Didier AugustoKarchev, NaoumVelhinho, JoséKarczewski, KrzysztofVeloz, TomasKarczmarek, PawełVelten, HermanoKardas, DariuszVenhuizen, Noortje J.Kärenlampi, PetriVentura, CarloKari, LeifVenturi, DanieleKarim, LutfulVera, TeresaKarimov, TimurVerde, FrancescoKarimullin, KamilVerdicchio, MarioKarlos, StamatisVerdoolaege, GeertKarperien, Audrey L.Verdu, SergioKarpiński, MikołajVerdú, SergioKarwasz, GrzegorzVergos, KonstantinosKarwowski, JacekVerousis, ThanosKassai, MiklosVerros, GeorgeKastner, RuthVersteegh, MarijnKastrup, BernardoVetro, CalogeroKateris, DimitriosVianna, Reinaldo OliveiraKatira, ParagVicencio, Rodrigo BarrazaKato, KentaroVicente, Rodriguez MontequinKatz, GarrettVidiella-Barrranco, AntonioKauf, SabinaVieira, AbelKavic, MichaelVieira, Marcus FragaKay, JamesVignes, MatthieuKay, Jim W.Vilcu, Gabriel EduardKaya, AlihanVilé, GianvitoKazakov, VladimirVillafaina, SantosKeeler, CynthiaVillecco, FrancescoKelbert, MarkVillmann, ThomasKeler, AndreasVilone, DanieleKeller, KarstenVining, Kyle HolmbergKellman, MichaelVinte, ClaudiuKenett, DrorVisintin, MonicaKennedy, Dallas C.Vitanov, Nikolay KKent, AdrianVitanov, Nikolay V.Keppens, ArnoVitányi, PaulKeshmiri, SoheilViterbo, EmanueleKesidis, GeorgeVitiello, GiuseppeKestler, Hans A.Vivo, PierpaoloKeziou, AmorVladimir, RoubtsovKhachay, MichaelVogel, Eugenio E.Khalifa, AmalVolkau, IharKhalifa, FahmiVolkovich, ZeewKhalil, AshrafVolodymyr, PalahinKhalili-Araghi, FatemehVolosencu, ConstantinKhalique, ChaudryVoloshynovskiy, SlavaKhan, AmirulVolosniev, ArtemKhan, FaisalVon Toussaint, UdoKhan, ImranVora, AnkitKhan, Tariq MahmoodVoulodimos, AthanasiosKhanh Le, Nguyen QuocVourdas, ApostolosKharel, RupakVourdoubas, JohnKharin, YuriyVranes, MilanKhorshidi, Hadi AkbarzadehVulpiani, AngeloKhosravy, MahdiVuruskan, AslihanKhovanov, IgorVyas, NikhilKiefer, AlexWachter-Zeh, AntoniaKim, Bom SooWacławczyk, MartaKim, CheonshikWadayama, TadashiKim, Cheorl-HoWagemakers, AlexandreKim, Eun-jinWagner, NathanielKim, HyejiWalkden, CharlesKim, HyunjuWalock, MichaelKim, IlkiWan, FengKim, Jong-ChanWan, KaiKim, Jong-MinWang, BohongKim, JungmuWang, BoxiangKim, NamWang, Chih-HungKim, Yong SinWang, DongKim, YongdaeWang, EdwinKim, Young-SikWang, GaigeKimovski, DragiWang, Gai-GeKing, MaxwellWang, HongKiourt, ChairiWang, JiangjiangKirichenko, LyudmylaWang, JianhuiKirillov, AlexanderWang, Jian-ShengKirmani, Syed N.U.A.Wang, JiayinKish, Laszlo B.Wang, JinKishore, RatnaWang, JingKitagawa, JiroWang, JunKiyono, KenWang, KangKjelstrup, SigneWang, KezeKlaassen, Chris A.J.Wang, Li-ChunKlapalova, AlenaWang, LigongKlasnja-Milicevic, AleksandraWang, Lin-ShuKlebanov, LevWang, MinKleidis, KostasWang, NingKlein, IngoWang, ShaoqingKlein, TonyWang, ShipingKlimenko, AlexanderWang, WendongKlimov, AndreiWang, Xiao-HuiKnopoff, DamianWang, XingyuanKobayashi, TeruyoshiWang, XuewenKoceva, Aleksandra RashkovskaWang, YawenKoch, TobiasWang, YixinKochańczyk, MarekWang, ZixiongKochar, SubhashWanke, PeterKocharovsky, VitalyWarne, DavidKocourek, AlesWarren, Patrick B.Koehler, MatthewWatanabe, KazuhoKoenig, Bryan L.Watanabe, ShunKogias, DimitrisWatrobski, JaroslawKolchinsky, ArtemyWeaver, MarkKolev, MikhailWeber, MelanieKollmann, WolfgangWei, FengyingKoltsov, SergeiWei, ShuangqingKomargodski, IlanWeinberger, NirKong, XiangxiongWeitschek, EmanuelKononovicius, AleksejusWen, Kun-LiKonstantakopoulos, Ioannis C.Wengerowsky, SoerenKontar, EduardWerle, SebastianKontogiannis, IoannisWettstein, MartinKonys, AgnieszkaWhitaker, AndrewKorabel, NickolayWhitlock, PaulaKorbel, JanWhitney, RobertKordov, KrasimirWibowo, SantosoKoronacki, JacekWibral, MichaelKoronovskii, AlexeyWichert, AndreasKorte, ArthurWidge, Alik S.Köse, SelçukWidom, MichaelKosiński, PiotrWiener, GerryKosloff, RonnieWiesner, KarolineKostadinov, DimcheWigger, MicheleKostina, VictoriaWilding, MartinKotelba, AdrianWilkinson, MichaelKotochigova, SvetlanaWillis, AshleyKotropoulos, ConstantineWilson, JulieKotsiantis, SotirisWilson, KatheryneKoubaa, AnisWilson, Stephen G.Koubiti, MohammedWimberger, SandroKoutmos, DimitriosWiseman, Howard M.Kovács, RóbertWiśniewski, PiotrKovács, SándorWitczak, PiotrKovalenko, Ilya G.Witkovsky, ViktorKowalski, GregoryWłodarczyk, ZbigniewKowalski, MarcinWohl, Christopher J.Kozaitis, SamuelWójcik, Grzegorz MarcinKozma, LászlóWolf, ChristianKrajewski, JarekWołk, KrzysztofKrajzewicz, DanielWollstadt, PatriciaKrakovská, AnnaWolpert, DavidKramer, BorisWong, Albert S.Y.Krapf, DiegoWong, Pak KinKrasil’shchik, JosephWong, Tzu-TsungKrasteva, VesselaWong, Wing-KeungKrause, AndrewWoo, JaeKrawec, Walter O.Wootters, WilliamKrishnan, PrasadWu, ChaofanKristoufek, LadislavWu, Hao-TianKrivoshein, AleksandrWu, Hsien-TsaiKrivovichev, VladimirWu, JianhuaKröker, IljaWu, JianZhongKrstacic, GoranWu, JingKrumhansl, CarolWu, JohnKruszewska, NataliaWu, Kuop JuiKtenidou, Olga-JoanWu, Shuen-DeKu, Cheng-YuWu, TailinKučera, ErikWu, XiaoYuKudryashov, BorisWu, YuKudryashov, NikolaiWüthrich, ChristianKugiumtzis, DimitrisWysocki, MarianKuhn, BerndXavier, Carolina RibeiroKujala, TuomoXavier, PradipKułakowski, KrzysztofXia, HuaKulikovskikh, IlonaXia, KelinKull, KaleviXiang, JiaweiKumar, Moses AshokXiao, YanghuaKumbhakar, ManotoshXiao, ZhuKunert-Graf, James M.Xie, LiangKung, Chien-ChunXin, BaoguiKunst, FloreXing, HaipengKuok, Sin-ChiXu, FeiKupczynski, MasrianXu, JunKurizki, GershonXu, LishengKurzynski, PawelXu, MengKuś, MarekXu, TianhuaKusaka, IsamuXu, YongKuschnerov, MaximYablonsky, Gregory S.Kutafina, EkaterinaYadav, RajeevKutner, RyszardYakovenko, VictorKuzilek, JakubYamagata, TakayukiKvalseth, TaraldYamakami, AkeboKvam, Peter D.Yamano, TakuyaKwak, Ho YoungYamauchi, TakashiKwapień, JarowslawYang, Cheng-YingKwek, Leong ChuanYang, Ching-YuKwon, HyukjoonYang, Chun-WeiKwon, Roy H.Yang, FanKwon, Seol-AYang, HongwenKwon, Soon-kakYang, LicaiKwon, YounghunYang, MinoYangKycia, RadosławYang, SamKynev, SergeyYang, Xiao-JunKyriakopoulos, KonstantinosYang, YaoqingLábár, János L.Yang, YongLabatut, VincentYang, YueLacki, JakubYang, YunLad, FrankYang, ZhenBiaoLadyman, JamesYannacopoulos, Athanasios N.Lagani, VincenzoYanofsky, Noson S.Lages, JoséYao, DongdongLaguna, HumbertoYasseri, TahaLahmiri, SalimYasunaga, TakeshiLai, ChinLunYazdanpanah, HamedLai, Kuan-TingYe, GuodongLai, YingxuYe, ZhuolinLaín, SantiagoYen, JianLaio, AlessandroYilmazkuday, HakanLamberti, Pedro WalterYing, ShihuiLambić, DraganYokomizo, NelsonLami, LudovicoYoo, Sung JinLampart, MarekYoon, Seong-MinLancho, AlejandroYoon, SoraLand, IngmarYoon, Tae JunLando, TommasoYordanova, KristinaLanfredi, MariaYoshimura, HiroakiLanga, Jose AYouk, HyunLanzetta, FrancoisYoung, DerekLao, YingjieYoung, Jean-GabrielLapenta, GiovanniYousefi, BardiaLapidoth, AmosYousif, Jabar H.Laplanche, GuillaumeYousof, Haitham M.Larecki, WieslawYoussef Moussa, Miled HassanLarentzakis, AndreasYoussri, Youssri H.Larocque, DenisYu, ChenglongLarrate, FrederiqueYu, LiguoLarson, JonasYu, MingchaoLashin, SergeyYu, ShaodeLaso, ManuelYu, ShujianLatif, Muhammad AmerYu, WeizeLau, EllenYuan, RuoshiLau, John W.Yuan, XiaoLau, Terry Shue ChienYue, JingLaurinec, PeterYue, YoufengLavacca, Francesco GiacintoYuen, Ka-VengLawless, Williams F.Yufik, Yan M.Lawrence, JayYukalov, VyacheslavLazarescu, MihaiYukalov, Vyacheslav I.Lazarovici, DustinYurekli, OsmanLe Ny, ArnaudZaburdaev, VasilyLe Page, ChristopheZachreson, CameronLe Sommer, NicolasZafar, AmadLéandre, RémiZagorowska, MartaLech, PiotrZaharia, MihaiLecomte, VivienZaidi, AbdellatifLee, Chou-YuanZakinyan, ArthurLee, Chyi LinZakrzewski, JakubLee, Dong-HoonZaky, Mahmoud A.Lee, HyunkwangZambrano, DavideLee, JaesungZambrano-Serrano, ErnestoLee, Jeong WooZanardi, PaoloLee, JohnZanni, LucaLee, Jong YunZanni-Merk, CeciliaLee, JulianZanón, José Luis VilarLee, KijoonZdravevski, EftimLee, KyeongjunZebrowski, Jan J.Lee, SeongwookZeleny, MartinLee, SeunggyuZeller, Camila BorelliLee, SkyZeng, XiangzeLee, SungjuZengler, KarstenLee, UnCheolZenil, Hectorleff, harveyZhan, KunLegnani, WalterZhang, DiLeister, WolfgangZhang, GuyueLeitão, JoãoZhang, HaoLeite Freire, IgorZhang, HaochunLemnaru, CameliaZhang, HongboLemus, EnriqueZhang, JiaxinLente, GáborZhang, JiazhongLentmaier, MichaelZhang, JunLenzi, Ervin K.Zhang, JunhuanLenzi, Ervin KaminskiZhang, JunlingLeón Espinoza, Edgar AlejandroZhang, LinfengLeonida, LeoneZhang, QiLeonski, WiesławZhang, QichunLepoitevin, MathildeZhang, SuLerga, JonatanZhang, TianweiLerma, ClaudiaZhang, WenyiLeszczuk, MikołajZhang, XianweiLevashov, Pavel R.Zhang, XiaoguangLevitan, JacobZhang, Xiao-YuLeviyang, SivanZhang, XueyingLeyuan, LiZhang, YanpengLeyvraz, FrancoisZhang, YinLeyvraz, FrançoisZhang, YuLezzi, Adriano M.Zhang, YudongLhotska, LenkaZhang, YupingLi, BinZhang, Zi-KeLi, ChengqingZhao, LiangLi, ChunbiaoZhao, Wan-LeiLi, HaoZHAO, YANLi, JieZhao, Yu-XiangLi, JunweiZhbanov, AlexanderLi, LinZhdankin, VladimirLi, MinZheng, MinzhangLi, NaipengZheng, ShuLi, PengZheng, Tong-XingLi, QiZheng, YuhuiLi, RuiZhou, JunhongLi, TaiyongZhou, LinLi, WangZhou, PengLi, WuchenZhou, YanengLi, Xiang-YiZhou, YongquanLi, XiantaoZhou, ZhiguoLi, XiaoboZhou, ZhuhuangLi, XiaocanZhu, BangzhuLi, XiaoliZhu, CongxuLi, XinghuaZhu, ZhenLi, XingjieZhu, ZhiqinLi, XiongZhuang, ShengchaoLi, YangminZieliński, WojciechLi, YiZierenberg, JohannesLi, YongboZietsch, BrendanLi, Yung-HuiZimont, Vladimir L.Li, YuxingZinovyev, AndreiLi, ZhilinZippo, Antonio GiulianoLi, ZuxingZivieri, RobertoLiao, HaoZlatić, VinkoLiao, HuchangZolfani, Sarfaraz HashemkhaniLiao, Jie-QiaoZotin, AlexeyLiao, LingminZou, JunLiau, Ben-YiZou, YajieLiaw, PeterZuin, MatteoLicata, IgnazioZullo, FedericoLicitra, GaetanoZuo, YiLillo-Saavedra, MarioZwirn, Hervé

